# Short-term outcome after transcatheter aortic valve replacement with a novel balloon-expandable valve

**DOI:** 10.1007/s12471-022-01738-z

**Published:** 2022-12-08

**Authors:** J. Halim, P. den Heijer, B. van den Branden, M. Meuwissen, J. Vos, B. Schölzel, A. IJsselmuiden

**Affiliations:** Department of Cardiology, Amphia Hospital Breda, Breda, The Netherlands

**Keywords:** Aortic stenosis, Transcatheter aortic valve replacement, Balloon-expandable valve

## Abstract

**Objectives:**

Transcatheter aortic valve replacement (TAVR) has been expanding rapidly with numerous transcatheter heart valve (THV) systems currently available. The Myval balloon-expandable (BE) valve (Meril Life Sciences Pvt. Ltd., India) is a novel THV system indicated for the treatment of patients with severe aortic stenosis. The primary objective of this study is to assess the safety and performance of the Myval BE valve.

**Methods:**

In this prospective single-centre study, 120 consecutive patients who underwent TAVR with the Myval BE valve were included. Clinical outcomes were evaluated at 30 days and 6 months using Valve Academic Research Consortium‑2 criteria. All-cause mortality, stroke, acute kidney injury, major vascular complications, moderate or severe paravalvular leakage (PVL) and need for a permanent pacemaker implantation (PPI) were investigated.

**Results:**

At 6‑month follow-up, all-cause death and cardiac death were seen in 5.8% and 0.8% of the patients respectively. Periprocedural stroke and need for PPI were both seen in 3.3% of the patients. Access-site-related vascular and bleeding complications were absent. Improved valve haemodynamics and no moderate to severe PVL could be seen at 30 days. An intermediate valve size was selected in 51% of the patients.

**Conclusions:**

The Myval BE valve demonstrates improved valve haemodynamics, absence of moderate to severe PVL and good safety outcomes at 6‑month follow-up with low cardiac death rate and acceptable rates of permanent pacemaker implantation and periprocedural stroke. Future randomised controlled trials will further establish the clinical utility of the Myval BE valve.

## What’s new

- The Myval BE valve is a novel transcatheter heart valve which provides additional valve sizes (intermediate and XL valve sizes) in addition to the conventional valve sizes aiming to minimise the risk for undersizing and oversizing.
- Good short-term safety and efficacy outcomes can be observed with the Myval BE valve with absence of moderate or severe paravalvular leakage and a low rate of permanent pacemaker implantation and cardiac death.
- A significant part (51%) of the patients received an intermediate valve size, indicating that there is a demand for additional valve sizes in order to facilitate precise device sizing.

## Introduction

Contemporary transcatheter heart valve (THV) systems for transcatheter aortic valve replacement (TAVR) are known to be associated with a low risk for periprocedural complications, favourable valve haemodynamics and better clinical outcomes [1–6]. Hence, TAVR has gone through a rapid evolution during recent years and is now considered indispensable in the treatment of severe aortic stenosis.

Importantly, randomised controlled trials have proven that TAVR is safe and efficacious in patients at high or intermediate surgical risk [7–13]. Recently, these results could also be reproduced in patients at low surgical risk [14, 15]. These positive findings have contributed to an increasing number of TAVR procedures being performed worldwide. Consequently, manufacturers have been focused on enhancing the technical aspects of their THV systems in order to improve clinical outcomes and lower the risk for adverse events, such as moderate to severe paravalvular leakage (PVL) and need for permanent pacemaker implantation (PPI). In addition, novel THV systems are being produced and given the opportunity to compete with currently well-known THV systems. One of these novel THV systems is the Myval balloon-expandable (BE) valve (Meril Life Sciences Pvt. Ltd., India).

The MyVal-1 study, the first-in-human prospective study, confirmed the safety and efficacy of the Myval BE valve [16]. From then on, a growing interest in the Myval BE valve can be observed with clinical data gradually expanding and two large-scale randomised controlled trials currently enrolling [17–23].

An important feature of the Myval BE valve is the availability of intermediate and XL valve sizes (in addition to conventional sizes), providing the operator a more comprehensive device size selection in order to lower the risk for undersizing or oversizing. We do, however, first need future studies to confirm the results of the MyVal-1 study and to elucidate whether the Myval BE valve is non-inferior to currently used THV systems.

The primary objective of this prospective study is to assess the safety and performance of the Myval BE valve in patients with severe aortic stenosis in a single heart centre in the Netherlands.

## Methods

### Study design

In this prospective single-centre study, we collected data from 120 consecutive patients who have undergone TAVR with the Myval BE valve. These patients had native, severe symptomatic aortic stenosis and were eligible for TAVR after Heart Team discussion. Patients with a bicuspid aortic valve were excluded. Surgical risk status was not taken into account for study inclusion. All patients gave written informed consent for the collection of their data within the scope of scientific research. Standard-of-care device size selection was done with a multi-detector computed tomography scanning according to the TAVR protocol using dedicated software (3mensio, Pie Medical Imaging, Maastricht, the Netherlands).

In all patients, a Myval BE valve was implanted in the Amphia hospital, Breda, the Netherlands between October 2019 and June 2021. In our centre, general anaesthesia and surgical cut-down of the femoral artery were considered standard of care. The transapical access route was seen as an alternative if femoral access was deemed unsuitable. After valve implantation, transoesophageal echocardiography was performed to evaluate the degree of PVL. Postdilatation with an increased balloon volume was performed if more than mild PVL was observed.

After TAVR, clinical follow-up took place at 30 days and 6 months. Echocardiographic follow-up was performed at 30 days.

### Myval THV system

The Myval BE valve is constructed in hybrid fashion using hexagonal cells with a large open cell design towards valve outflow zone (or the upper end) accounting for 53% of the expanded frame height and two rows of closed cell design towards valve inflow zone (or the lower end) accounting for 47% of the expanded frame height [23]. As a result, preservation of coronary flow and higher radial strength are achieved. The frame itself is composed of an alloy of nickel and cobalt. To minimise PVL, the lower end of the frame is covered internally and externally with a polyethylene terephthalate cuff. Furthermore, an anti-calcification treatment has been added (AntiCa, Meril Life Sciences Pvt. Ltd., India) to the tri-leaflet valve that is made out of bovine pericardium. The THV is crimped on the Navigator balloon-catheter delivery system before insertion in the sheath. Hereafter, excellent flexibility is provided by the Navigator balloon-catheter delivery system. During deployment, the distal and proximal part of the balloon will expand first to allow enhanced stability. The hexagonal valve frame, upon crimping, will appear as an alternative dark-light band-like pattern during fluoroscopy. With this pattern, valve implantation is made easy and takes place at annular level.

An important feature of the Myval BE valve is the availability of additional sizes. More specifically, besides the traditional sizes (20, 23, 26 and 29 mm) intermediate sizes (21.5, 24.5 and 27.5 mm) and extra-large sizes (30.5 and 32 mm) are available. Lastly, a 14F Python sheath can be used for all available valve sizes.

### Study endpoints

Clinical outcomes were evaluated at 30 days and 6 months using Valve Academic Research Consortium‑2 criteria. We investigated all-cause mortality, stroke, myocardial infarction, acute kidney injury, major vascular complications, moderate or severe PVL, conduction system disturbances resulting in a new PPI. Echocardiographic outcomes were assessed at 30 days.

### Statistical analysis

Descriptive statistics were used for statistical analysis. Continuous variables were shown as mean ± standard deviation, whereas categorical variables were shown as frequencies and percentages. All analyses were conducted with SPSS v.26 (IBM, Chicago, IL, USA).

## Results

### Baseline characteristics

In this study, we included 120 patients treated with the Myval BE valve. The baseline characteristics are shown in Tab. 1 and 2. The mean age of our study population was 80.2 ± 6.3 years. Of the patients, 53% were men and the mean EuroSCORE II was 4.0 ± 2.8. A pacemaker was already present in 8% of the patients. The mean aortic valve area was 0.77 ± 0.18 cm^2^. In 13% of the patients, the left ventricle was moderately or severely reduced.

**Table 1:** Clinical baseline characteristics

	*n* = 120 *N* (%) or mean ± SD
Age	80.2 ± 6.3
Male	64 (53)
BMI	28.2 ± 4.6
EuroSCORE II	4.0 ± 2.8
NYHA class III or IV	23 (38)
Diabetes mellitus	43 (36)
Hypertension	85 (71)
Coronary artery disease	56 (47)
Previous CABG	13 (11)
Chronic kidney disease	42 (35)
Cerebrovascular disease	24 (20)
Peripheral vascular disease	17 (14)
COPD	19 (16)
Atrial fibrillation	39 (33)
Prior pacemaker	10 (8)
RBBB	12 (10)
LBBB	11 (9)

*BMI* body mass index, *NYHA* New York Heart Association, *CABG* coronary artery bypass grafting, *COPD* chronic obstructive pulmonary disease, *RBBB* right bundle branch block**, ***LBBB* left bundle branch block

**Table 2:** Imaging baseline characteristics

	*n* = 120 *N* (%) or mean ± SD
*Echocardiographic measurements*	
LVEF ≤ 40%	16 (13)
AV area, cm2	0.77 ± 0.18
AV mean gradient, mm Hg	37.4 ± 13.5
Moderate or severe mitral regurgitation	18 (15)
*MDCT measurements*	
Annulus perimeter, mm	78.2 ± 7.2
Annulus area, mm^2	467.7 ± 83.8
Maximum annulus diameter, mm	27.7 ± 3.7
Mean annulus diameter, mm	24.6 ± 2.2
Minimum annulus diameter, mm	21.3 ± 2.2
Perimeter derived diameter, mm	24.9 ± 2.3
Area derived diameter, mm	24.3 ± 2.2
Maximum aorta ascendens, mm	32.3 ± 3.1
Minimum femoral artery diameter, mm	6.3 ± 1.4

*LVEF* left ventricular ejection fraction, *AV* aortic valve, *MDCT* multidetector computed tomography

### Procedural data

**Fig. 1:**
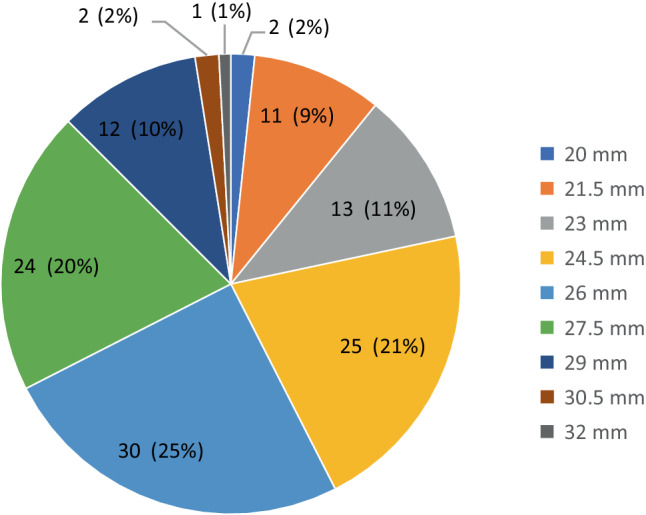
Valve size selection

In 107 of the 120 patients (89.1%), the femoral artery was the preferred access route. In the other patients (*n* = 13), a transapical TAVR was performed. Predilatation and postdilatation were performed in 4.1% and 2.5% of the patients respectively. Valve embolisation after deployment was seen in two patients (1.7%). In these two patients, a second valve implantation with an Evolut THV was necessary. An overview of valve size selection is shown in Fig. 1. In 51% of the patients, an intermediate valve size was implanted.

### Clinical outcomes

Clinical outcomes are shown in Tab. 3. At 30-day follow-up, three patients (2.5%) had died. Two of these three patients died as the result of a periprocedural complication. In the first patient we observed a right coronary artery obstruction after valve deployment resulting in cardiac arrest. In the second patient, the THV could not pass the aortic valve due to severe calcification despite proper predilatation. Device retrieval in the sheath was then unsuccessful, after which valve deployment took place in the abdominal aorta. This patient died due to an abdominal aorta rupture occurring after deployment. The third patient died because of pneumonia during hospitalisation.

**Table 3:** Clinical outcomes

Clinical outcomes	30 days *n* = 120 *N* (%)	6 months *n* = 120 *N* (%)
All-cause death	3 (2.5)	7 (5.8)
Cardiac death	1 (0.8)	1 (0.8)
Annular rupture	0 (0)	–
All stroke	4 (3.3)	7 (5.8)
Myocardial infarction	1 (0.8)	1 (0.8)
Acute kidney injury	5 (4.2)	–
Moderate or severe paravalvular leakage	0 (0)	–
New permanent pacemaker implantation	4 (3.3)	4 (3.3)
Vascular and access-site-related complications	0 (0)	–

At 30-day follow-up, stroke was observed in four patients (3.3%). In all four patients, stroke was observed shortly after TAVR. A PPI was needed in four patients (3.3%), of which two patients received an intermediate valve size. After TAVR, acute kidney injury was seen in five patients (4.2%). Importantly, complete recovery was seen at discharge in all patients. In all patients, access-site-related vascular and major bleeding complications were absent.

Between 30-day and 6‑month follow-up, cardiac death was absent in all patients. Four patients died nonetheless during this follow-up period. In two patients, a cerebrovascular accident occurred which quickly led to death. The other two patients died because of lymphoma and rapid deterioration of dementia. Moreover, a cerebrovascular accident was seen in one other patient with good neurological recovery. Importantly, no other major adverse events were documented at 6‑month follow-up.

### Echocardiographic outcomes

**Fig. 2:**
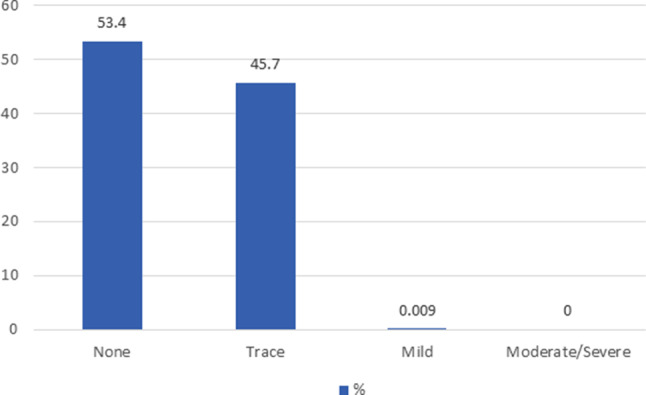
Distribution of the degree of PVL at 30 days. *PVL* paravalvular leakage

**Fig. 3:**
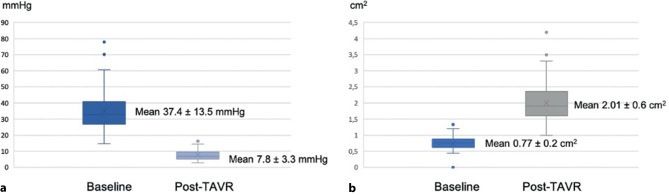
Aortic valve haemodynamics at baseline compared with post-TAVR. *TAVR* transcatheter aortic valve replacement

In our study, moderate to severe PVL was absent at 30 days in all patients. The distribution of the degree of PVL is illustrated in Fig. 2. After TAVR, improved valve haemodynamics could be seen with a decrease of aortic valve mean gradient from 37.4 ± 13.5 mm Hg to 7.8 ± 3.3 mm Hg and an increase of aortic valve area from 0.77 ± 0.2 cm^2^ to 2.01 ± 0.6 cm^2^ (Fig. 3).

## Discussion

In this prospective single-centre study, we consecutively enrolled 120 patients with native severe aortic stenosis who all underwent TAVR with the Myval BE valve. Our principal findings are that: 1) the Myval BE valve is associated with an acceptable rate of periprocedural complications and good safety outcomes at 6‑month follow-up; 2) successful implantation of the Myval BE valve is seen in 98.3% of the patients with improved valve haemodynamics and absence of moderate to severe PVL; 3) the availability of additional valve sizes proves to be useful with 51% of the patients receiving an intermediate valve size.

The performance of the Myval BE valve was first investigated in the MyVal-1 study (*n* = 30) with good safety and performance outcomes at 12-month follow-up [16].

Hereafter, a multicentre prospective study (*n* = 416) was conducted wherein propensity score matching was performed to compare the Myval BE valve with the Sapien 3 valve [17]. At 30-day follow-up, no significant differences in early safety and clinical efficacy were shown. Importantly, no moderate to severe PVL and a lower PPI rate (5.8% vs 15.5%; *p* = 0.02) were observed with the Myval BE valve. The lower PPI rate should, however, be carefully interpreted due to absence of adjustment for baseline conduction disturbances and central electrocardiographic analysis.

Moreover, lower transvalvular mean gradients (*p* < 0.001) and a lower rate of moderate to severe patient-prosthesis mismatch were observed with the Myval BE valve (0% vs 3.9%; *p* = 0.043), which persisted after correction for predilatation and postdilatation. Notably, a significant part of the patients received an intermediate valve size (44.6%). It can, therefore, be hypothesised that the intermediate valve sizes play an important role in this interesting finding. In our study, an intermediate valve size was selected in 51% of the patients. Kawashima et al. confirmed this finding in a large group (*n* = 1115) of patients treated with the Myval BE valve in which an intermediate valve size was implanted in 42% of the patients [18]. We can, therefore, conclude that there is demand for a more precisely scaled device size matrix with the inclusion of intermediate valve sizes.

Until now, in case of borderline anatomy (annulus area on the borderline between two device sizes) optimal device sizing was achieved with underfilling or overfilling the balloon [24, 25]. While this strategy has shown to be effective, there is still concern of its risks and its potential negative effect on leaflet function and valve haemodynamics.

In our study, good short-term outcomes with the Myval BE valve were established. In particular, absence of moderate to severe PVL and a low rate of PPI associated with this newer generation THV were confirmed. Nevertheless, periprocedural stroke still occurred in 3.3% (*n* = 4) of the patients, which is slightly higher than what we have come to expect in contemporary times. Important to note is that valve embolisation was seen in one of the four patients necessitating a second valve implantation, which could have potentially increased the risk for periprocedural embolisation and stroke. Moreover, absence of access-site-related vascular complications should be put in perspective. Due to the extremely low vascular complication rate, a more controlled surgical approach of the femoral artery is still being applied in our centre. We acknowledge that this is not considered “standard of care” in most centres where a percutaneous approach is preferred. Additionally, valve embolisation (*n* = 2), which is a rare complication in TAVR, was caused by loss of capture due to rapid ventricular pacing on the guidewire during valve deployment. Hereafter, we decided to systematically perform rapid ventricular pacing with a temporary pacemaker lead in the right ventricle.

We believe that the availability of an expanded and more calibrated device size matrix of the Myval BE valve allows for a more tailored device size selection taking into account the patient’s anatomy and preserving the geometry of the prosthesis. This important feature can potentially lower the risk for procedure-related adverse events, such as moderate to severe PVL, need for PPI and annular rupture. While available data with regard to the Myval BE valve are promising, randomised controlled trials (COMPARE-TAVI and the LANDMARK trial) are essential to elucidate whether the Myval BE valve is indeed non-inferior to current THV systems [22].

## Limitations

This study has several limitations. First, the relatively small sample size and short follow-up period. That is why we cannot provide any information on long-term safety and performance outcomes of the Myval BE valve. Secondly, the observational nature of this study. Future, large randomised controlled trials are a necessary next step to provide us with more information on whether or not the Myval BE valve can compete with contemporary THV systems.

## Conclusion

The Myval BE valve confirms good safety and performance outcomes at 6‑month follow-up. Absence of moderate to severe PVL and acceptable rates of PPI and periprocedural stroke could be observed. Future randomised controlled trials will elaborate us further on the clinical utility of the Myval BE valve.
